# Clinical Applications of Remote Ischemic Preconditioning

**DOI:** 10.1155/2012/620681

**Published:** 2012-02-14

**Authors:** Kristin Veighey, Raymond J. MacAllister

**Affiliations:** ^1^University College London Centre for Nephrology, Royal Free Campus, Rowland Hill Street, London NW3 2PF, UK; ^2^University College London Centre for Clinical Pharmacology, Rayne Institute, 5 University Street, London WC1E 6JF, UK

## Abstract

Ischemia-reperfusion injury is a composite of damage accumulated during reduced perfusion of an organ or tissue and the additional insult sustained during reperfusion. Such injury occurs in a wide variety of clinically important syndromes, such as ischemic heart disease and stroke, which are responsible for a high degree of morbidity and mortality worldwide. Basic research has identified a number of interventions that stimulate innate resistance of tissues to ischemia-reperfusion injury. Here, we summarise the experimental and clinical trial data underpinning one of these “conditioning” strategies, the phenomenon of remote ischemic preconditioning.

## 1. Introduction

Ischemia-reperfusion injury underpins the damage of myocardial infarction, stroke, and other conditions complicated by interruption of the blood supply to tissues. Strategies to limit the duration of ischemia have achieved substantial health gains in myocardial infarction and, to a lesser degree, stroke. However door-to-needle times have probably reached the minimum that is possible in many health-care delivery systems, so further reduction in morbidity and mortality from IR injury will require strategies to increase tissue tolerance to ischemia or reduce damage that occurs on reperfusion. One such approach is ischemic preconditioning, and its variant remote ischemic preconditioning, the subject of this paper.

## 2. Types of Ischemic Preconditioning

Ischemic preconditioning (IPC) describes the phenomenon whereby transient, brief periods of ischemia confer protection against a subsequent prolonged and injurious period of ischemia. There are a number of ways in which preconditioning can be induced. Local preconditioning occurs when the preconditioning stimulus is applied to the same organ or tissue that will subsequently sustain the ischemic injury. Remote ischemic preconditioning refers to a stimulus applied to a distant organ or tissue, which then protects against index ischemia. For example, the preconditioning stimulus might be suprasystolic blood pressure inflations on an arm or leg, which then confer myocardial protection against subsequent ischemia. Postconditioning occurs when there is staged reperfusion, for example, in the setting of balloon angioplasty. Its variant perconditioning occurs when the conditioning stimulus is applied during ischemia.

## 3. Ischemic Preconditioning

Ischemic preconditioning was first described in 1986, when Murry et al. demonstrated that in the dog, brief episodes of ischemia (4 cycles of 5-minute occlusion followed by reperfusion) of the circumflex artery reduced the extent of infarction induced by subsequent prolonged occlusion of that vessel [1]. This protection expired after a few hours, but subsequent studies indicated that it recovered approximately 24 hours later, this second phase of protection lasting for up to a further 72 hours [2–4]. Despite the length of time that has elapsed since the discovery of IPC, detailed exposition of its mechanism, and evidence that the biological processes operate in humans, IPC has never progressed to detailed clinical investigation. This was largely due to the logistics of inducing preconditioning ischemia in vital organs (such as the heart or brain) in advance of a more prolonged insult (e.g., that would lead to myocardial infarction or stroke).

## 4. Remote Ischemic Preconditioning

A major breakthrough in clinical applicability of preconditioning protection came with the discovery that ischemic preconditioning also had a systemic protective phenotype. This facet, termed remote ischemic preconditioning (RIPC), resulted in protection from ischemia-reperfusion injury at sites remote from those undergoing the preconditioning stimulus. This was first described in the setting of experimental coronary artery occlusion, where preconditioning one vascular territory of the heart effected protection in adjacent tissue that had not undergone any preconditioning ischemia [5]. Interorgan protection was confirmed by the observation that preconditioning stimuli applied to the small bowel [6] or kidney [7] reduced infarct size in the heart. As was the case for IPC, further studies established that the time course of protection caused by RIPC was also biphasic. The demonstration that RIPC could be activated merely via brief periods of limb ischemia simplified the logistics of inducing ischemic preconditioning in animals and humans [8]. Moreover, RIPC activated by limb ischemia protected from experimental IR injury in humans. Together these observations framed the conditions that have led to a large number of clinical trials of RIPC in patients.

## 5. Mechanisms of Tissue Protection of IPC and RIPC

During ischemia, anaerobic metabolism predominates and ATP production decreases. There is insufficient available energy to maintain cell membrane pump activity, antioxidant defences, pH and calcium homeostasis, and mitochondrial integrity. These and other consequences of ischemia inevitably lead to cell death, unless blood flow is restored. Though reperfusion with oxygenated blood is essential for any tissue salvage, the sudden influx of oxygen leads to the formation of reactive oxygen species. A key event in cell death is mitochondrial permeability transition, a phenomenon that occurs when the mitochondrial permeability transition pore (mPTP) becomes permeable to molecules of 1500 kDa or smaller. This leads to a rapid influx of small molecules, mitochondrial swelling, and subsequent cell death [8].

IPC activates three main salutatory pathways, the cyclic guanosine monophosphate/cGMP-dependent protein kinase (cGMP/PKG) pathway [9], the reperfusion injury salvage kinase (RISK) pathway [10], and the survivor activating factor enhancement (SAFE) pathway [11]. There is a degree of overlap, in particular where the pathways converge on the mitochondrion [12]. Here, the potassium-dependent ATP (K_ATP_) channel is activated with evidence that this leads to closure of the mPTP. IPC also initiates a complex genomic and proteomic response that underpins the late phase of protection. This includes antiapoptotic and anti-inflammatory gene transcription, likely to be responsible for the second window of protection [13, 14].

Triggers in the initial cascade recruit early mediators (such as protein kinase C (PKC), tyrosine kinase, phosphatidylinositol 3-kinases (PI3K), protein kinase B (PKB or Akt) [15], mitogen-activated protein kinases (MAP1/2 or MEK1/2), extracellular signal-regulated kinases (Erk1/2), and janus kinase (JAK)), which activate transcription factors (such as signal transducer and activator of transcription proteins (STAT1/3), nuclear factor kappa-light-chain-enhancer of activated B cells (NF*κ*B), activator-protein-1 (AP-1), nuclear factor-like 2 (Nrf2), and hypoxia-inducible-factor-1*α* (HIF-1*α*)). Later phase protection requires synthesis of inducible nitric oxide synthase (iNOS), heat shock proteins (HSPs), or cyclooxygenase-2 (COX-2). These then act locally via the mPTP or K_ATP_ channels to induce a state of cardioprotection [16].

## 6. Mechanism of the Systemic Spread of Protection

Evidence for involvement of a humoral factor in mediating systemic spread is supported by the observation that protection can be transferred by the transfusion of serum from a rabbit that has undergone ischemic preconditioning to one which has not [17, 18]. This factor appears to be heat stable and has been shown to be dialysable and of less than 15 kD [19, 20]. In some studies it is blocked by opioid antagonists, including naloxone [21, 22].

Neurogenic mechanisms have also been explored using autonomic ganglionic blockade. In a rat myocardial infarction model hexamethonium abolished protection by RIPC achieved by mesenteric artery occlusion (MAO) but had no effect on myocardial IPC. Cardioprotection was absent when MAO was sustained throughout the study, indicating that reperfusion in the small intestine was essential to activate the neurogenic pathway [6]. The autonomic ganglion blocker trimethaphan has also been shown to inhibit remote ischemic preconditioning in a human model [23]. In the rabbit, sympathetic nerve activity increases when RIPC is induced using renal ischemia, consistent with a particular role for the adrenergic component of the autonomic system in this species [24].

Noradrenaline has been implicated; however, studies are conflicting about its potential role. Administration of noradrenaline has been shown to induce preconditioning in animal models [25]; however, studies differ as to whether alpha-adrenoceptor blockers such as prazosin inhibit preconditioning [25, 26]. Noradrenaline levels were not seen to be increased in the serum of preconditioned rabbits, which when transfused conferred preconditioning, leading to doubts about a role in the transfer of protection [18].

Sensory nerves have also been implicated in spread of protection. Intramesenteric bradykinin has been demonstrated in animal models to stimulate local sensory nerves, resulting in RIPC-like protection that is abolished by hexamethonium [27]. This suggests a pathway involving sensory nerves and the autonomic nervous system. However, a recent human healthy volunteer study demonstrated that the bradykinin-2 inhibitor HOE-140 had no effect on RIPC [28], so as ever mechanisms might be different in humans. Calcitonin-gene-related peptide (CGRP), a neurotransmitter in capsaicin-sensitive sensory nerves (CSSNs), has also been implicated [29, 30]. CGRP has been reported to increase systemically after RIPC, and pretreatment with capsaicin (to deplete CSSN) blocks RIPC [31].

The nonselective adenosine antagonist 8-(p-sulfophenyl)theophylline (8-SPT) has been shown in a rabbit [24, 32, 33] and rat [34] model to abolish the protective effects of remote ischemic preconditioning. Adenosine might therefore be one step in a complex pathway, though not itself the circulating factor.

The humoral and neuronal pathways may work in series to spread protection systemically. In the rat, release of the dialysable humoral factor is prevented by hindlimb denervation [35].

**Figure 1:**
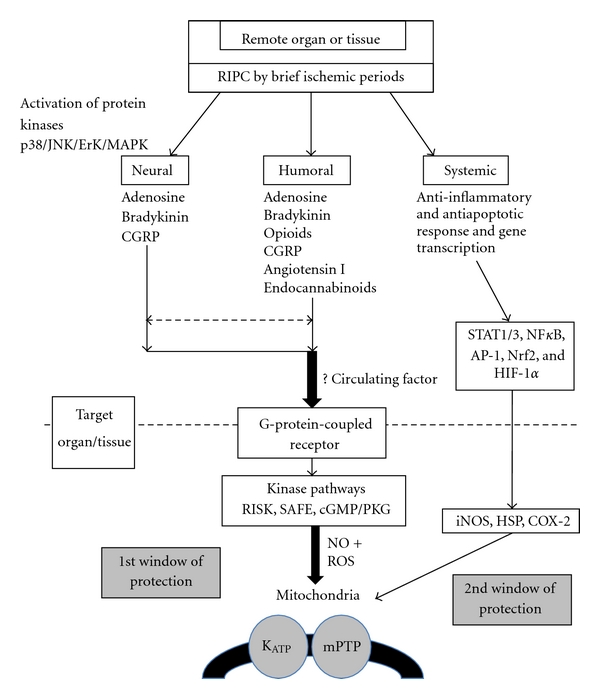
proposed mechanisms of remote ischemic preconditioning.

The mechanisms of tissue protection and systemic spread have been summarised in Figure 1. 

## 7. Mechanism of IPC and RIPC in Humans

The development of a vascular model of human IR injury has facilitated the investigation of the mechanism of IPC and RIPC. In this model the arm is made ischemic for 20 minutes, and this safely induces a transient period of endothelial dysfunction in the conduit and resistance vessels. Endothelial assessment has been made using ultrasound of conduit vessels to measure flow-mediated dilatation or forearm plethysmography to characterize the response of resistance vessels to endothelium-dependent agonists. In this model, vascular smooth muscle function is preserved. Endothelial dysfunction is largely prevented if the ischaemic period is preceded by brief, repeated periods of ischaemia ipsilaterally (IPC) [36] and contralaterally (RIPC) [37, 38], with two phases of protection [38]. Administration of K_ATP_ channel blockers prevents IPC in healthy volunteers, and IPC is mimicked by K_ATP_ channel opening drugs [39, 40]. A number of studies suggest that IR injury is dependent on increased oxidative stress [41, 42], making it possible that IPC and RIPC stimulate antioxidant defences [43]. Regarding the systemic spread of protection, ganglionic blockade inhibits both phases of RIPC [6, 38], though as yet it is unclear which component of the autonomic system is responsible. Dialysate of human plasma from volunteers who have undergone RIPC reduces IR injury in vitro in an opiate-dependent manner, and this supports activation of opiate pathways in humans [20]. However, the relative contributions of the neuronal and humoral pathways remain to be determined.

## 8. Clinical Trials: Current Status

Although initial clinical trials in this area focussed on the application of remote ischemic preconditioning in ischemic cardiac disease, interest has broadened to other areas of potential clinical benefit, including acute kidney injury, stroke, and transplantation.

A table summarising clinical trials in remote ischemic preconditioning to date is shown (Table 1).

### 8.1. Cardiac Surgery

The first clinical trial of remote ischemic preconditioning was in 2000, when 8 patients undergoing coronary artery bypass grafting (CABG) were randomised to receive either ischemic preconditioning (forearm cuff inflated to 300 mmHg for 2 cycles of 3 minutes) or control. The study demonstrated an increase in lactate dehydrogenase (LDH) in the preconditioned group, which was attributed to an ability to maintain anaerobic metabolism in preconditioned cells [44].

Following this, a randomised controlled trial of RIPC in the setting of pediatric surgery (37 patients) for congenital cardiac defects demonstrated that 4 cycles of 5 minutes of lower limb ischemia prior to surgery were effective in reducing troponin levels, postoperative inotropic requirements, and airways resistance at 6 hours [45]. A study of arm preconditioning in simple congenital cardiac defects (ventricular septal defect repair) demonstrated an improvement in lung compliance and a decrease in cardiac enzymes (LDH, CK, and troponin I) and cytokines (IL-6, IL-8, IL-10, and TNF-*α*) in the preconditioned group. In this study, those randomised to the preconditioning group received preconditioning at 24 hours and 1 hour before surgery, to utilise both early and late phases of protection [46].

Subsequently in 2007, Hausenloy et al. demonstrated a reduction in troponin T levels in patients randomised to receive ischemic preconditioning (3 cycles of 5-minute forearm cuff inflation to 200 mmHg after induction of anaesthesia) prior to coronary artery bypass grafting [47]. In both these trials, the patients underwent cross-clamp fibrillation; however, Venugopal et al., in 2009, also demonstrated a reduction in troponin T following remote ischemic preconditioning in patients undergoing cold blood cardioplegia [48].

However, in 2010, Rahman et al. published a larger single-centre double-blind randomised controlled trial in which 162 patients undergoing CABG were randomised to receive either 3 × 5-minute cycles of upper limb cuff inflation to 200 mmHg (separated by 5-minute reperfusion) or placebo (in which the cuff was inflated on a “dummy arm”). In this study there was no difference in troponin release between the 2 groups [49]. There was also no difference in cardiac performance, inotrope requirement, echocardiographic function, arrhythmia protection, or renal and lung outcomes.

The RICO trial (the Effect of Remote Ischemic Conditioning on Atrial Fibrillation and Outcome) is ongoing to examine the effects of remote ischemic preconditioning, remote ischemic postconditioning, or remote ischemic pre- and postconditioning on the development of atrial fibrillation on holter monitor within 72 hours after coronary artery bypass grafting [50]. This will help to define if there is any clinical benefit of combining preconditioning strategies.

A current multicenter double-blind randomised controlled trial, “Effect of Remote Ischemic Preconditioning on Clinical Outcomes in Patients Undergoing Coronary Artery Bypass Graft Surgery” (ERICCA), is currently underway to investigate whether RIPC improves one-year cardiovascular outcomes and reduces acute kidney injury (AKI) in the setting of cold-blood cardioplegia CABG. This trial aims to recruit 1610 patients, randomised to either RIPC or sham-RIPC.

### 8.2. Percutaneous Coronary Intervention (PCI) for Acute Myocardial Infarction (AMI)

In 2006, Iliodromitis et al. investigated whether RIPC by 3 cycles of 5-minute ischemia applied to both arms would attenuate the inflammatory response in elective single vessel PCI with coronary stenting. They in fact demonstrated an increase in CK-MB, troponin I, and CRP in the preconditioned group and postulated that RIPC increased the inflammatory response [51].

Subsequently, Hoole et al., in 2009, in a study of 242 patients undergoing elective PCI demonstrated that RIPC (3 cycles of 5-minute forearm cuff inflation to 200 mmHg) prior to PCI attenuated procedure-related cardiac troponin I (cTnI) release [52]. Diabetics and hypertensives were included and also benefited. However, in a separate study the same group showed that there was no beneficial effect on left ventricular dysfunction during coronary balloon occlusion in single vessel coronary disease [53].

Also of note, Bøtker et al. demonstrated the potential for prehospital use of remote ischemic *perconditioning* in the setting of AMI (4 cycles of 5-minute forearm cuff inflation and deflation, delivered in the ambulance). In a trial of 333 patients, they demonstrated an improvement in myocardial salvage index (%) at 20 days after primary PCI in the group randomised to receive preconditioning [54]. In a substudy of the same patients, remote ischemic conditioning delivered before hospital seemed to result in modest improvement in LV function in high-risk patients prone to develop large myocardial infarcts [55].

### 8.3. Vascular Surgery

In open abdominal aortic aneurysm (AAA) repair, 82 patients were randomised to receive either RIPC (two cycles of intermittent cross-clamping of the common iliac artery with 10-minute ischemia followed by 10-minute reperfusion) or none. RIPC reduced the absolute risk of myocardial injury, myocardial infarction, and renal injury [56]. Another study in the same clinical scenario, in which 51 patients were randomised to sequential common iliac artery cross-clamping as the conditioning stimulus or none, did not demonstrate any improvement in renal outcome indices (urinary retinol binding protein (RBP) and albumin : creatinine ratio (ACR)) [57]. The same group, however, demonstrated lower levels of RBP and albumin : creatinine ratio following surgery in the preconditioned group in the setting of endovascular AAA repair (EVAR) [58].

A UK-based trial of remote ischaemic preconditioning in the setting of elective abdominal aortic aneurysm repair is currently underway in Bristol.

### 8.4. Brain and Neurological Injury

A pilot clinical trial of 70 patients undergoing carotid endarterectomy, randomised to remote ischemic preconditioning with 10 minutes of ischemic applied to each leg showed a numerical but not statistical reduction in saccadic latency deteriorations (the primary neurological outcome). There was no significant difference between cardiac endpoints [59].

However, in a trial in 40 adult cervical spondylotic myelopathy patients undergoing elective decompression surgery there was a significant reduction in markers of ischemic neurological injury (serum S-100B and neuron-specific enolase) in the RIPC group. Patients randomized to RIPC received 3 × 5-minute arm cycles. Postsurgical recovery at 7 days, 1, and 3 months after surgery (evaluated using a Japanese Orthopaedic Association scale) was higher in the preconditioned group [60].

In stroke, the “New Acute Treatment for Stroke—The Effect of Remote Perconditioning” has recently closed to recruitment and results awaited. This trial has examined the utility of remote ischemic preconditioning using arm ischemia in the ambulance prior to hospital admission and thrombolysis. The primary endpoint in this study is salvage index, measured on diffusion-weighted T2 MRI.

### 8.5. Acute Kidney Injury (AKI)

IR injury contributes to the majority of AKI. Common causes of AKI such as sepsis, surgery, or drugs, for example, NSAIDS, ACE inhibitors, are all essentially wholly or in part due to ischemia-reperfusion injury, secondary to hypotension or reduced renal blood flow. AKI is responsible for morbidity and increased mortality in hospital admissions; however, its onset is often unpredictable and patients present when AKI is already manifest. Therefore, use of RIPC to protect against acute kidney injury has most widely been documented in the setting of surgery. In this setting, however, the acute injury is complex and multifactorial in nature.

Secondary analysis from two trials in elective coronary artery bypass grafting has demonstrated a reduction in AKI in nondiabetic patients randomised to RIPC (three 5-minute cycles of forearm ischemia). However, the numbers were small, there were more concomitant aortic valve replacements in the RIPC group, the analysis was post hoc, and, although there were more episodes of AKI in the nonintervention group, this was all AKI stage 1. [61] In a separate study of lower limb preconditioning in the setting of cardiac surgery requiring cardiopulmonary bypass, AKI was reduced in the RIPC group (47% versus 20%). All AKI was either stage 1 or 2 [62]. A recently published trial of leg preconditioning in children undergoing surgery for complex congenital cardiac disease found no evidence that preconditioning protected renal function. End points were development of acute kidney injury, initiation of dialysis, plasma creatinine, estimated glomerular filtration rate, plasma cystatin C, plasma and urinary neutrophil gelatinase-associated lipocalin, and urinary output [63]. A similar study in adults again demonstrated no evidence of benefit in renal protection following complex cardiac surgery; however, a reduction in CK-MB was observed [64].

Trials examining the effects of preconditioning on AKI in the setting of AAA surgery have been detailed above.

#### 8.5.1. Solid Organ Transplantation

The ultimate renal IR injury occurs in renal transplantation, and in the case of live donor transplantation this has a predictable time of onset. Loukogeorgakis et al. have previously demonstrated an almost 20% improvement in eGFR (Modification of Diet in Renal Disease (MDRD) formula) after 2 years in children who underwent RIPC before live donor renal transplantation. Both donor and recipient received 3 cycles of 5-minute forearm ischemia-reperfusion, 24 hours prior to transplantation. This was a small randomised controlled trial in 20 patients; however, a larger, 400-pair multicentre randomised controlled trial (Renal Protection against Ischemia Reperfusion in Transplantation (REPAIR)) is currently underway. A trial of RIPC in the setting of cadaveric renal transplantation is being undertaken by Bøtker et al. The RIPCOT trial (Remote Ischemic Preconditioning in Abdominal Organ Transplantation), which utilises lower limb remote ischemic preconditioning in the setting of deceased donor liver, kidney, or pancreas transplantation, and RIPC before abdominal surgery trial in those undergoing abdominal, large bowel, pancreatic, and hepatic surgery are also currently underway.

## 9. Conclusion

Remote ischemic preconditioning harnesses a powerful innate protective mechanism against ischemic injury. The mechanism is as yet not fully elucidated; however, it has shown promise in clinical trials. No trial has yet been large enough to demonstrate an effect of RIPC to reduce the incidence or impact of clinically relevant consequences of IR injury. However, large adequately powered studies will report within the next 2-3 years. These trials will either endorse the clinical usefulness of ischemic preconditioning or consign it to the laboratory.

## Figures and Tables

**Table 1:** Clinical trials to date in remote ischemic preconditioning.

Year	*n*	Author	Clinical setting	Conditioning protocol	Results
2000	8	Günyaydin	Coronary artery bypass grafting (CABG)	Arm 2 × 3 min	**↑**Lactate dehydrogenase (LDH) in preconditioned group
2005	12	Chan	Clipping of cerebral aneurysm following subarachnoid haemorrhage	Proximal artery 2 min catheter occlusion, 30 min reperfusion	**↓**tissue hypoxia (slower decline in pO2 and pH)
2006	41	Iliodromitis	Elective single vessel percutaneous coronary intervention (PCI) and stenting	Both arms 3 × 5 min	Increased troponin I, CK-MB, and C-reactive protein (CRP)
2006	37	Cheung	Paediatric surgery for congenital cardiac defects	Leg 4 × 5 min	**↓**troponin T, **↓**post-op inotrope requirements, **↓**airway resistance
2007	82	Ali	Elective open abdominal aortic aneurysm repair	Internal iliac cross-clamp 2 × 10 min	**↓**absolute risk of myocardial injury/infarction (troponin I) and renal injury (creatinine)
2007	57	Hausenloy	Elective CABG	Arm 3 × 5 min	**↓**troponin T
2008	165	Faries	Balloon occlusion during carotid angioplasty and stenting	Temporary balloon occlusion of ICA	Decreased Glasgow Coma Score (GCS) seen on initial balloon inflation not observed when blood reinflated
2009	45	Venugopal	CABG (cold-blood cardioplegia) +/− valve replacement	Arm 3 × 5 min	**↓**troponin T
2009	242	Hoole	Elective PCI	Arm 3 × 5 min	**↓**troponin I, reduces ischaemic pain during PCI
2009	42	Hoole	Elective PCI for single vessel coronary artery disease	Arm 3 × 5 min	No effect on left ventricular dysfunction (conductance catheter/Doppler/dobutamine stress echocardiogram)
2009	40	Walsh	Endovascular AAA repair	Both legs 10 min ischaemia	Reduced urinary retinol binding protein and albumin : creatinine ratio
2010	70	Walsh	Carotid endarterectomy	Both legs 10 min ischaemia	Fewer saccadic latency deteriorations (did not reach statistical significance)
2010	78	Venugopal	CABG	Arm 3 × 5 min	Post hoc analysis of 2 previous studies. Decreased Acute Kidney Injury (AKI) on Acute Kidney Injury Network (AKIN) criteria
2010	162	Rahman	CABG (on-pump)	Arm 3 × 5 min	No difference in trop T/cardiac performance/inotrope requirement/echo function/arrthymias/renal or lung outcomes
2010	120	Wagner	CABG with cold crystalloid cardioplegia	Arm 3 × 5 min	**↓**troponin I; tramadol administration increased troponin
2010	51	Walsh	Open infrarenal AAA repair	Sequential common iliac clamping	No differences in renal outcomes (urinary retinol binding protein (RBP) and albumin : creatinine ratio (ACR))
2010	60	Wenwu	Ventricular septal defect repair	Arm 3 × 5 min; 24 h and 1 h before surgery	Reduced cytokines and cardiac enzymes, upregulation of heat shock protein (HSP) 70; improved lung compliance
2010	40	Hu	Elective decompression surgery for adult cervical spondylotic myelopathy	Arm 3 × 5 min	Reduced markers of ischaemic neuronal injury S-100B and neuron-specific enolase; improved recovery rate on functional score
2011	120	Zimmerman	Cardiac surgery with cardiopulmonary bypass	Leg 3 × 5 min	Reduced relative risk of AKI on AKIN criteria
2011	113	Pedersen	Paediatric surgery for correction of complex cardiac defects	Leg 4 × 5 min	No effect on development of AKI, urine output, or urinary biomarkers
2011	76	Choi	Complex valvular heart surgery with CPB	Leg 3 × 5 min	No effect on creatinine, cystatin C, or neutrophil-gelatinase-associated lipocalin (NGAL), estimated glomerular filtration rate Decreased CK-MB at 24 hours
2011	242	Munk	Primary PCI for acute MI	Arm 4 × 5 min, prehospital	Modest improvement in LV systolic function (not significant)
